# Colonic Recurrence of Clear Cell Renal Cell Carcinoma 13 Years After Nephrectomy: A Case Report

**DOI:** 10.1155/crom/6822885

**Published:** 2026-10-26

**Authors:** Ivo Klarin

**Affiliations:** ^1^ General Hospital Zadar Zadar Zadar County Croatia; ^2^ University of Zadar Department of Health Studies Zadar Zadar County Croatia

## Abstract

The colon is a rare site of solitary metastasis from renal cell carcinoma. It may present years after curative resection of the primary tumor, usually with colonic obstruction or gastrointestinal bleeding. We report the case of an 82‐year‐old man with colon metastasis 13 years after curative nephrectomy for renal cell carcinoma.

## 1. Introduction

Renal cell carcinoma accounts for about 3% of all cancers in adults and is the third most common urological tumor (after prostate and bladder carcinoma) [1]. It is more common in men [2]. The classic triad of hematuria, flank pain, and a palpable mass is present in approximately 17% of patients. Most cases are asymptomatic and diagnosed incidentally [1]. Despite radical surgical resection, about one‐third of patients will have metastases after nephrectomy. The most common sites of metastasis are the lungs, bones, liver, and brain [3]. Metastases to the digestive system are very rare, especially to the colon [3]. Fewer than 30 cases of colonic metastasis from renal cell carcinoma have been reported to date, the first case in 1991 [4, 5]. We present a case of symptomatic (bleeding) metastasis of clear cell renal carcinoma to the colon, which underwent successful curative surgery.

## 2. Case Report

An 82‐year‐old man with a past medical history of essential hypertension was diagnosed with clear cell renal cell carcinoma in 2011 and underwent radical left nephrectomy (the tumor measured 6.5 cm in diameter and infiltrated the renal capsule, extending into the perirenal adipose tissue. It involved the walls of large blood vessels but spared the adrenal gland and the resected ureteral margin. There was an absence of nodal disease. Histology results were as follows: clear cell type, ISUP Grade 2; Fuhrman nuclear Grade 2). Follow‐up PET/CT imaging in 2017 revealed pulmonary metastasis and a solitary metastasis in the left psoas muscle. The patient received sunitinib (50 mg, 4 weeks on/2 weeks off) from December 2017 to August 2018. It was chosen because of its local availability at the time. A favorable response, with regression of both metastatic lesions, was achieved, and the patient subsequently underwent video‐assisted thoracoscopic surgery (VATS) with left lower lobectomy for the pulmonary metastasis, along with resection of the solitary left psoas muscle metastasis (histology: clear cell type and ISUP Grade 2). The International Metastatic RCC Database Consortium (IMDC) score was 2. Sunitinib was discontinued following disease progression and subsequent complete surgical resection of the pulmonary and psoas metastases. After complete metastasectomy and achievement of no evidence of disease (NED) status, the treating oncologist elected to discontinue systemic therapy and continue surveillance, taking into consideration the potential toxicity of sunitinib and the clinical practice at that time. Follow‐up CT scans performed between 2018 and 2024 demonstrated no evidence of recurrent or measurable disease after metastasectomy.

In December 2024, the patient developed intermittent hematochezia. ECOG was 1. Abdominal CT revealed a localized tumor mass in the splenic flexure (Figure 1). Colonoscopy confirmed the presence of the lesion (Figure 2), and surgical resection was performed. A left hemicolectomy with splenectomy was carried out. Thirty‐seven centimeters of colon, omentum, and regional lymph nodes were resected. The tumor measured 7 × 4 × 2 cm. Histological examination confirmed metastatic clear cell renal carcinoma, identical to the primary tumor (the immunohistochemical profile showed diffuse positivity for PAX8, CD10, CAIX, RCC marker, and pan‐cytokeratin [AE1/AE3], with negative staining for CK7, CK20, CDX2, SATB2, and TTF‐1) (Figure 3). No tumor involvement was found in the resected lymph nodes.

**Figure 1:**
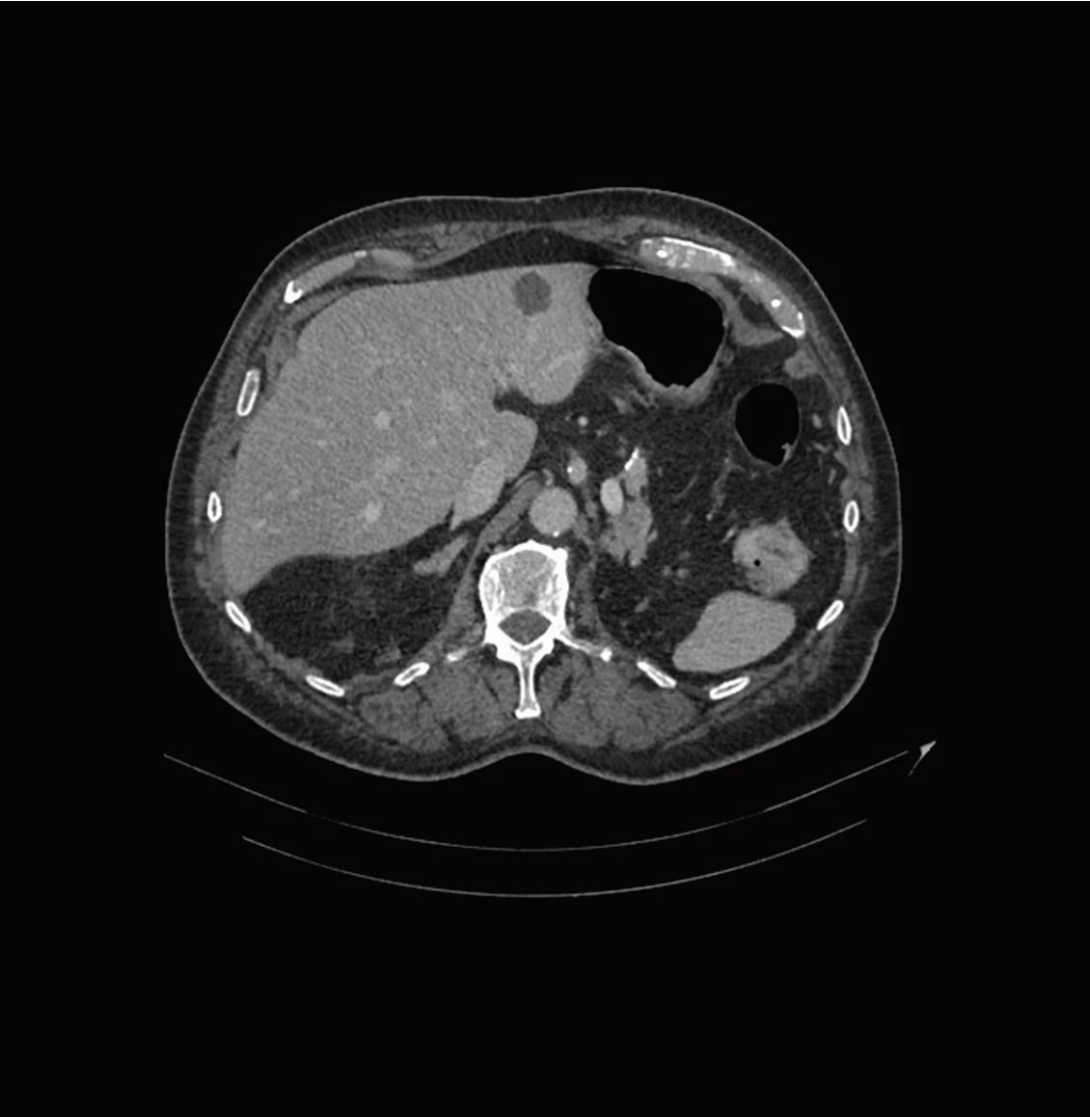
Contrast‐enhanced computed tomography demonstrating tumor mass 3 cm in diameter at the splenic flexure of the colon. No other suspicious lesions are seen.

**Figure 2:**
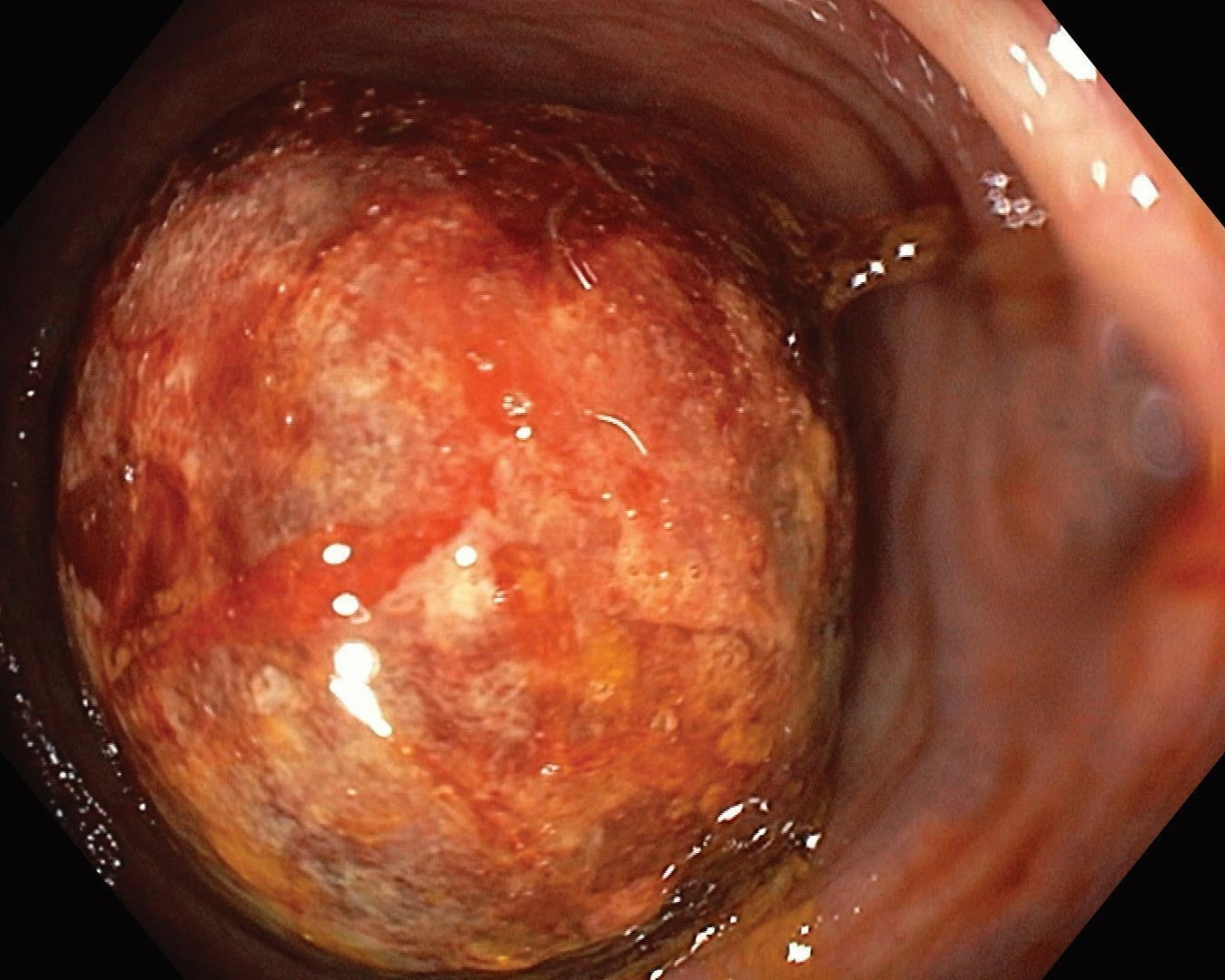
Colonoscopy showing tumor mass at the splenic flexure of the colon.

**Figure 3:**
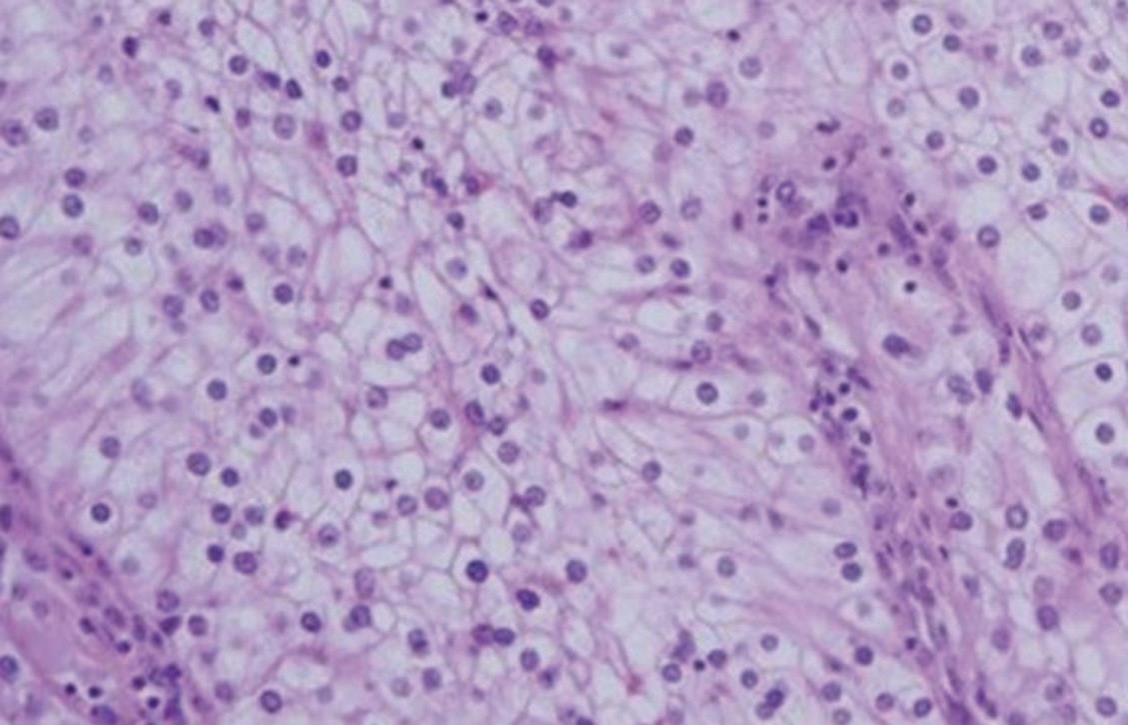
Histopathology of colonic mass demonstrating tumor cells with clear cytoplasm and atypical nuclei on H&E staining at ×20 magnification.

The postoperative course was uneventful, and the patient was discharged 2 weeks after surgery. Immunotherapy with nivolumab (240 mg every 2 weeks) was initiated in January 2025 in the absence of measurable disease and is ongoing. Nivolumab monotherapy was selected based on the treating oncologist′s clinical judgment, taking into consideration the patient′s previous treatment with a tyrosine kinase inhibitor. The patient remains without evidence of measurable disease on follow‐up imaging.

## 3. Discussion

Metastases of renal cell carcinoma to various organs have been widely reported [6]. However, metastases to the colon are extremely rare [7]. A review of the literature has described fewer than 30 cases [5, 8–11]. Most reported patients presented with symptoms, including bowel obstruction or gastrointestinal bleeding and associated with disseminated disease [12]. After curative nephrectomy, metastases usually appear within 3 years. Relapse of the disease after 5 years is rare, in less than 10% of patients, but is typical for colon metastases [13]. Patients with late disease recurrence generally have a better prognosis. Despite the lack of randomized prospective studies, there is a widely accepted consensus that surgical resection is the method of choice for these patients, especially if complete (R0) resection can be achieved [14]. Given the high rate of disease recurrence, follow‐up is an extremely important part of the treatment of renal cell carcinoma. There are recommendations for routine follow‐up for the first 5 years after radical nephrectomy, but there are no clear guidelines after that period [15]. Given the possible late occurrence of disease recurrence, postoperative follow‐up may need to be extended to 15 years. In patients presenting with gastrointestinal symptoms, colonoscopy with biopsy should be performed whenever feasible.

## Funding

No funding was received for this manuscript.

## Disclosure

The author has read and approved the final version of the manuscript, had full access to all of the data in this study, and takes complete responsibility for the integrity of the data and the accuracy of the data analysis. The author affirms that this manuscript is an honest, accurate, and transparent account of the study being reported; that no important aspects of the study have been omitted; and that any discrepancies from the study as planned have been explained.

## Ethics Statement

Ethical approval is not required for this study in accordance with local or national guidelines. All procedures followed were in accordance with the ethical standards laid down in the 1964 Declaration of Helsinki and its later amendments.

## Consent

Written informed consent was obtained from the patient for publication of this case report and any accompanying images.

## Conflicts of Interest

The author declares no conflicts of interest.

## Data Availability

The data that support the findings of this study are available on request from the corresponding author. The data are not publicly available due to privacy or ethical restrictions.
